# A Complex Presentation: Psychosis in a Patient Diagnosed With Lennox-Gastaut Syndrome

**DOI:** 10.7759/cureus.65010

**Published:** 2024-07-20

**Authors:** Gurraj Singh, Gurtej Gill, Satwant Singh, Nikhita S Roshan, Akshita Lalendran, Sasidhar Gunturu

**Affiliations:** 1 Psychiatry, Bergen New Bridge Medical Center, Paramus, USA; 2 Psychiatry, BronxCare Health System, New York, USA; 3 Psychiatry and Behavioral Sciences, Interfaith Medical Center, Brooklyn, USA; 4 Neurology, Father Muller Medical College, Mangalore, IND; 5 Psychiatry and Behavioral Sciences, BronxMedcare Hospital, New York, USA

**Keywords:** breakthrough seizure, seizure activity, psychosis, childhood epilepsy, lennox-gastaut syndrome

## Abstract

Lennox-Gastaut syndrome (LGS) is a form of severe childhood epilepsy, with most children experiencing seizures before reaching the age of eight. Typically, patients have multiple types of seizures, making an accurate diagnosis challenging. While it can be secondary to other causes, often, it is idiopathic. Over time, children develop cognitive impairment, leading to intellectual disability. The mainstay of treatment and management is seizure control. However, management remains challenging due to the complexity of the syndrome, as it is associated with multiple seizure types, intellectual deterioration, and other psychiatric comorbidities. We present the case of a 19-year-old male diagnosed with LGS and treated with various available therapies, who demonstrated multiple breakthrough seizures, significant neurocognitive disabilities, and behavior challenges. Additionally, the patient displayed psychotic features of auditory hallucinations, aggression, and attempts at self-mutilation, a rare clinical presentation in LGS.

## Introduction

Lennox-Gastaut syndrome (LGS) is a severe developmental epileptic encephalopathy characterized by generalized seizures in a high frequency of multiple varieties that occur in children between the ages of one and eight [1]. The International League Against Epilepsy defines LGS as having three characteristic features, namely, (a) multiple types of drug-resistant seizures with onset before 18 years (one of which must include tonic); (b) cognitive and often behavioral impairments, which may not be present at seizure onset; and (c) diffuse slow spike-and-wave and generalized paroxysmal fast activity on electroencephalography (EEG) [2,3]. LGS primarily originates more in the frontal lobe than other brain regions [1,4,5]. In LGS, different types of seizure can be observed. The most recurring seizure types include tonic seizures, primarily at night, atonic, and atypical absence seizures [6]. Children may present with status epilepticus, showing prolonged or repeated seizures in 60% of the cases, with a mortality rate between 3% and 7% [2,7].

Among childhood epilepsy cases, LGS has a prevalence of 3-10% and is approximately 1.5-2 times more common in male (61.5%) than in female (38.5%) patients [2,8]. Etiologies vary, ranging from a variety of genetic abnormalities, cortical lesions, and neurocutaneous disorders (e.g., tuberous sclerosis complex (TSC)) to underlying unknown etiologies in about 25% of patients.

Current data suggests that loss of function and missense mutations in the *CACNA1A* gene, which encodes a specific calcium channel alpha A1 subunit involved in mediating synaptic neurotransmitter release, might be responsible for some of the epileptic encephalopathies such as LGS [5,9,10]. These patients also show a spectrum of cognitive dysfunction in addition to psychomotor delay, attention-deficit hyperactivity disorder, autism spectrum disorder, and intellectual disability [9]. Several severe infantile epilepsy syndromes often evolve into LGS; however, a good differentiating factor is that most children with LGS have developmental impairment before the onset of seizures. Repetitive assessment for LGS criteria can help distinguish it from other types of seizure syndromes [3]. A considerable number of patients (20-60%) exhibit delayed development at the onset of LGS. By the fifth year from the onset of the syndrome, the percentage of patients experiencing cognitive impairments escalates to 75-95% [11-13].

LGS can quickly become a medical emergency, and managing these patients can be clinically challenging. We present a case that highlights a patient with LGS exhibiting psychotic features of auditory hallucinations, in addition to aggression, and attempts at self-mutilation, which is not commonly seen in LGS. We also provide information on managing the complexities of treating such a patient.

This case was presented as a poster at the American Psychiatric Association (APA) 2022 Annual Conference on May 22, 2022.

## Case presentation

A 19-year-old male presented to the emergency department (ED) with recurrent seizures after endorsing hearing voices and threatening self-mutilation. Emergency medical services (EMS) documented recurrent tonic-clonic seizure activity lasting over 20 minutes upon their arrival. According to the ambulance report, the patient had attempted to convey to his family that he was hearing “voices and sounds” before the seizures began while also threatening to self-mutilate his face with a blade. The seizures ceased after EMS administered two doses of lorazepam.

The patient’s past medical history included LGS, challenges with impulse control, autism, and intellectual disability. At age 10, the patient had undergone partial brain resection surgery to address recurring seizures, as narrated by his mother. Specific details regarding the resection were not obtained at the time.

In the ED, the patient exhibited combative behavior and agitation and expressed anger by punching objects while talking to himself. This behavior was consistent with collateral information from the patient’s mother, who reported that the patient had poor impulse control and episodes of aggression toward his “voices.” The mother also noted that the patient began exhibiting combative behavior following his brain resection surgery, which only worsened with age.

Following his ED admission, the patient was psychiatrically hospitalized. He appeared incoherent and irritable, demonstrating limited communication, and being unable to convey the events leading to his admission. He could not express the content of the auditory hallucinations or communicate effectively whether he presented with delusions or disorganized thoughts. However, during his hospital stay, the patient continued to experience episodes of breakthrough tonic-clonic seizures. Further medical evaluation was unremarkable, including frequent neurological examinations, toxicology labs, comprehensive metabolic panel, medication review, and electrolyte levels. The patient was routinely evaluated during his extended admission.

The previous diagnosis of LGS was consistent with the patient’s clinical findings, including a history of early-onset seizures, a past surgical history of brain resection at age 10, continued treatment-resistant seizures, and intellectual disability. An EEG for slow spike-and-wave patterns was attempted but was unsuccessful due to repeated seizure activity.

The patient was started on oral levetiracetam, clobazam, zonisamide, lacosamide, brivaracetam, and lorazepam for seizure control. Additionally, risperidone was initiated to address the positive psychotic symptoms. With the addition of the antipsychotic, the patient appeared to respond less to internal stimuli, with a noticeable reduction in irritability. The patient still exhibited infrequent breakthrough seizures lasting more than 15 minutes, with gargled breath sounds and apparent respiratory distress.

Once medically stabilized and with subsided seizure activity, the patient no longer displayed combative behavior or intent to self-mutilate. During his hospitalization, he received physical, occupational, and speech therapy. He was discharged after being deemed at his “baseline” by his family. Due to the patient’s limited verbal communication, a detailed history and complete assessment of his psychotic features, including delusions and disorganized thoughts, could not be obtained. Following discharge, no follow-up appointments were pursued by the patient or his family.

## Discussion

LGS is considered a severe form of childhood epileptic disorder, making it difficult for physicians and families to manage. Seizures start at a young age, with multiple types of seizures [14] perplexing the initial diagnosis from various pathological etiologies.

There are three leading categorical causes of LGS. They include structural causes (i.e., congenital brain malformation and acquired brain injury), metabolic disorders, and genetic-related causes [15]. Genetic causes (i.e., gene mutations) are recognized as a cause of early-onset and severe epilepsy, resulting in irreversible structural changes in the brain. This is similar to the intractable and variable seizures seen in TSC [16].

Management difficulties lead to long-term effects on cognition and behavior. The mainstay of treatment focuses on seizure control with an additional focus on high-level psychosocial intervention to support the development and behavioral needs of the patient. Despite adequate seizure control, patients can still display significant cognitive impairment within the first five years of diagnosis [2]. In addition to cognitive impairment, patients experience physical stresses due to repeated seizures and sleep disturbances [17]. Our patient was diagnosed with LGS for several years, with surgical intervention in addition to antiepileptic drug (AED) therapy for seizure control, yet was exhibiting several behavioral and psychiatric abnormalities, along with significant cognitive dysfunction.

The presence of behavioral and psychotic symptoms in our patient, while uncommon, is not surprising. Although the use of antipsychotics for the treatment of aggression in LGS is not routine, cases have been described in the past where patients with LGS and psychomotor agitation were successfully treated with risperidone, sodium valproate, lamotrigine, and clobazam [18]. The use of benzodiazepines, especially clobazam, for LGS has, however, been associated with aggression [19].

Irreversible structural brain changes, a known cause of LGS, often coincide with psychiatric disorders. In literature, a strong relationship between brain changes, primarily in the prefrontal cortex, and positive psychotic symptoms is often highlighted, with MRI evidence [20]. Prior cases of LGS and psychiatric comorbidities are often presented with schizophrenia, but none with the unique findings of positive psychotic features along with combative behavior and self-mutilation. These signs may point to psychiatric issues beyond cognitive impairment and developmental delay.

Additionally, despite multiple AEDs, it was difficult to control the seizure activity in the patient, with repeated breakthrough seizures. Seizure management in LGS is challenging due to the prevalent treatment-resistant seizures and the evolving nature of the disease, with multiple types of seizures clouding the treatment algorithms. Various AEDs, surgical procedures, and dietary modifications (i.e., ketogenic diet) have been used to gain control over an otherwise complex syndrome [14,21]. It is essential to note that while seizure control is critical in management, it often requires multiple therapeutic agents, which has its challenges. AEDs can provide some symptomatic relief from seizures, but not without the risk of side effects from polypharmacy, which include sedation, neurotoxicity, and paradoxical deterioration in seizure control [20,22]. Due to these side effects, LGS patients often require continuous support. A coordinated interprofessional team approach can improve patient outcomes and enhance quality of life. While limited information is available regarding the efficacy of current management strategies, outcomes for these patients remain poor, with a reported mortality rate of 3-7% over 8-10 years of follow-up [14].

The role of genetic factors is being explored and studied for further understanding and therapeutic advancements in LGS. The goal of such research is to eventually identify specific gene sequences that can be targeted to help control the symptoms associated with LGS using precision medicine. One such gene that has been evaluated is *CACNA1A* [23], used in controlling the synthesis of a protein called Cav2.1, which mediates fast communication between nerve cells [24]. In one study, specific gene sequencing identified *CACNA1A* as one of the pathogenic mutant genes present in epileptic syndromes [25]. *CACNA1A* mutations have been implicated in both pure epilepsy and a spectrum of epilepsy phenotypes based on the molecular sub-region of the mutations [26]. While genetics are considered crucial in many epileptic syndromes, specific genes have been identified in only a small number of cases [23].

The current study suggests a pressing need for further research into the connection between LGS and psychiatric conditions. The goal is to identify specific genetic factors leading to utilizing gene-directed therapies to help patients with LGS and other severe epileptic syndromes.

## Conclusions

The management of LGS presents complex challenges, particularly with the co-occurrence of psychiatric and behavioral symptoms. Effective management requires a multi-layered approach, incorporating AEDs to address intractable seizures and antipsychotics for any underlying psychotic features, if present. Although genetic research offers promise for improving LGS management, specific causative genes remain largely unidentified. This study highlights the necessity for further research into LGS, especially regarding the management of patients with potential psychiatric comorbidities.
